# Pediatric Upper Extremity Replantation: Courage in the Face of a Life-altering Injury

**DOI:** 10.1097/GOX.0000000000001766

**Published:** 2018-07-13

**Authors:** Wesley N. Sivak, M. Asher Schusterman, Lorelei J. Grunwaldt

**Affiliations:** From the Department of Plastic Surgery, University of Pittsburgh, Pittsburgh, Pa.

## Abstract

**Background:**

Pediatric plastic surgeons perform reconstructive surgeries for various congenital, oncologic, and traumatic injuries.

**Methods:**

Our Children’s Hospital of Pittsburgh of University of Pittsburgh Medical Center (UPMC) Plastic Surgery team was tasked to care for a young man who suffered a proximal humeral amputation of his dominant upper extremity.

**Results:**

A multidisciplinary team collaborated throughout his entire acute care and postoperative course, guiding treatment and care in effort to maximize function of his replanted extremity.

**Conclusions:**

This case report details the patient’s unique journey and highlights his determination and courage to return back to a normal life.

## Abstract 2

Supplemental Digital Content is available in the text.

## CASE STUDY

On November 8, 2015, an otherwise healthy 12-year-old male suffered a traumatic amputation of his dominant right upper extremity while working on his family’s farm. His coat sleeve caught in the spinning drive shaft that was powering a piece of heavy equipment, avulsing his arm at the level of the upper humerus. He was transferred via helicopter from the scene to Children’s Hospital of Pittsburgh of UPMC and arrived in stable condition. Given the severity of his injury, he was intubated in the trauma bay and taken emergently to the operating room. Both the plastic and orthopedic surgery teams were called in to evaluate the patient at that time. The patient’s other injures included a nondisplaced scapular fracture and separated shoulder. Aside from minor abrasions, there were no injuries to the arm distal to the amputation site, although it had suffered a severe stretch injury. After a discussion with the orthopedic surgery team, the decision was made to proceed with replantation, given the patient’s age and stable medical condition.

First, thorough debridement followed by osteosynthesis of the humerus was performed. Shortening osteotomies, 1.5 cm in total, were required to facilitate tension-free apposition of unrepaired structures. Microsurgical repair of the brachial artery and cephalic vein reestablished vascular flow. Musculocutaneous, radial, and median nerves were identified and repaired; the ulnar nerve was irreparably damaged. Avulsed segments of the biceps, brachialis, deltoid, and triceps were repaired with intramuscular sutures; the resulting skin defect was left open. Finally, forearm fasciotomies were performed. Ischemia from time of injury to revascularization was 6 hours.

## PEDIATRIC UPPER EXTREMITY REPLANTATION

Continued refinement of microsurgical technique has led to increasing success rates with replantation over the years.^1^ Broader inclusion criteria for pediatric replantation, together with the greater technical demands of the repair and the less favorable mechanism of pediatric amputations (ie,—crush-avulsion) yield a lower survival rate of the replanted part than in adults.^2^ However, the superior nerve and soft-tissue regenerative capacity of children appears to favor better functional outcomes. But issues of appearance and a developing self-image in a child transcend range of motion, sensation, and strength variables reported in the literature. Therefore, it remains imperative that microsurgical salvage always be attempted whenever possible in pediatric upper-extremity amputations.^3^

## ACUTE MANAGEMENT AND POSTOPERATIVE RECOVERY

Recovery was not easy for our patient but proceeded smoothly without any unexpected returns to the operating room; there were no subsequent issues with infection or wound healing. Postoperative monitoring in the intensive care unit included hourly neurovascular checks around the clock for several days—further stress after the injury and subsequent surgery. He later returned to the operating for skin grafting and fasciotomy closure. He remained on prophylactic antibiotics under the guidance of infectious disease physicians, given the severity and mechanism of the injury. He was only in the hospital 3 weeks before his discharge back to his home in rural Pennsylvania, almost 80 miles from the Children’s Hospital of Pittsburgh on Thanksgiving Day. Those 3 weeks would have taken their toll on anyone, but despite all he had been through he never lost hope.

**Fig. 1.**
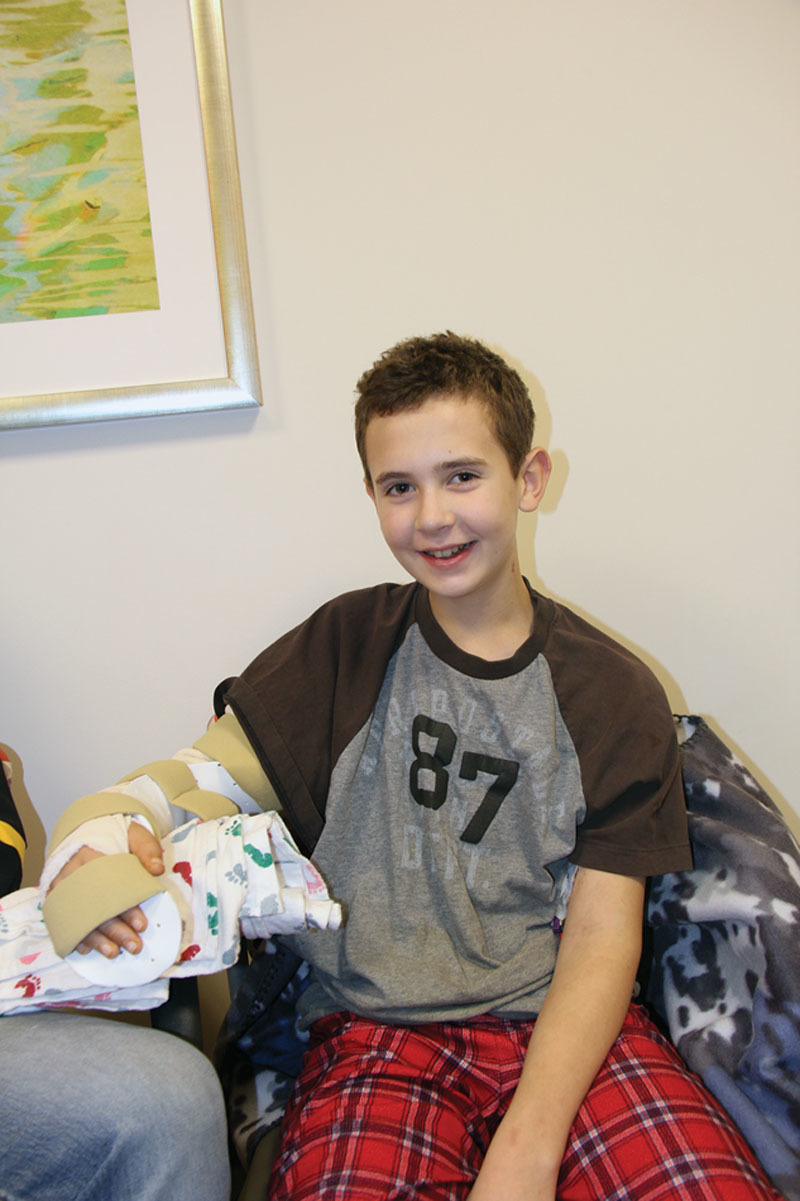
Six weeks out from right upper extremity replantation—his arm remained immobilized in a custom splint.

**Fig. 2.**
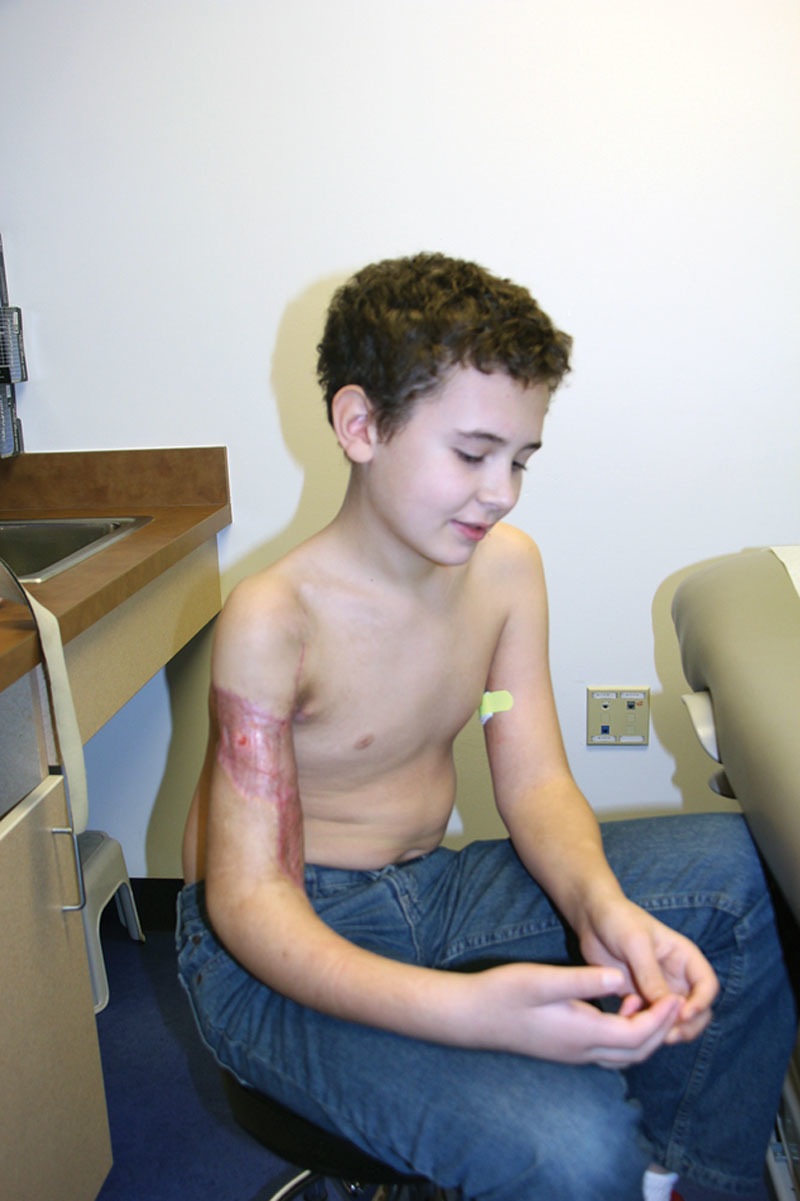
Three months out from right upper extremity replantation, beginning to show signs of reinnervation of physical examination.

**Fig. 3.**
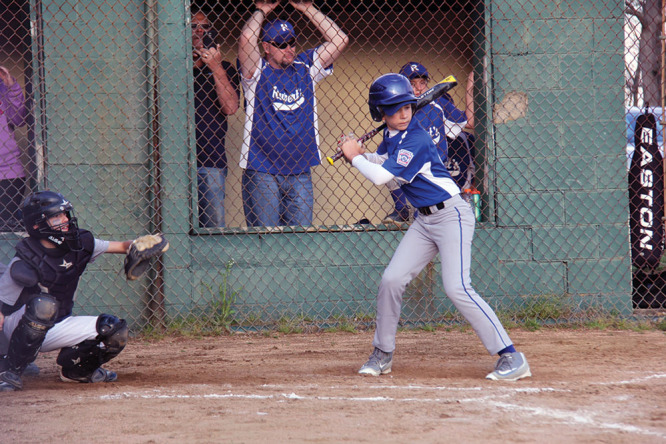
At bat during the little league baseball home opener 1-year following replantation.

The local media was made aware of this story immediately following his injury, and there was a strong outpouring of support for our patient that continues to the present time. At 6 weeks after surgery, despite all he had been through his spirits remained high and his resolve stronger than ever (Fig. 1). He endured intense physical and occupational therapy sessions, always relying on his own internal resolve. The atrophic soft tissues of his right arm slowly recovered and by 3 months had regained sensation down to the level of the mid-forearm (Fig. 2). By that time, he had also regained full shoulder movement, and elbow flexion against gravity. Rather than be discouraged, our patient was motivated to work even harder with hopes of rejoining his little league baseball team and perhaps even pitching once again. With unwavering dedication and focus, he has begun to show major improvement. At over a year out from the injury, he was back to playing baseball and had flickers of active movement in both his wrist and thumb (Fig. 3).

## CONCLUSIONS

**Fig. 4.**
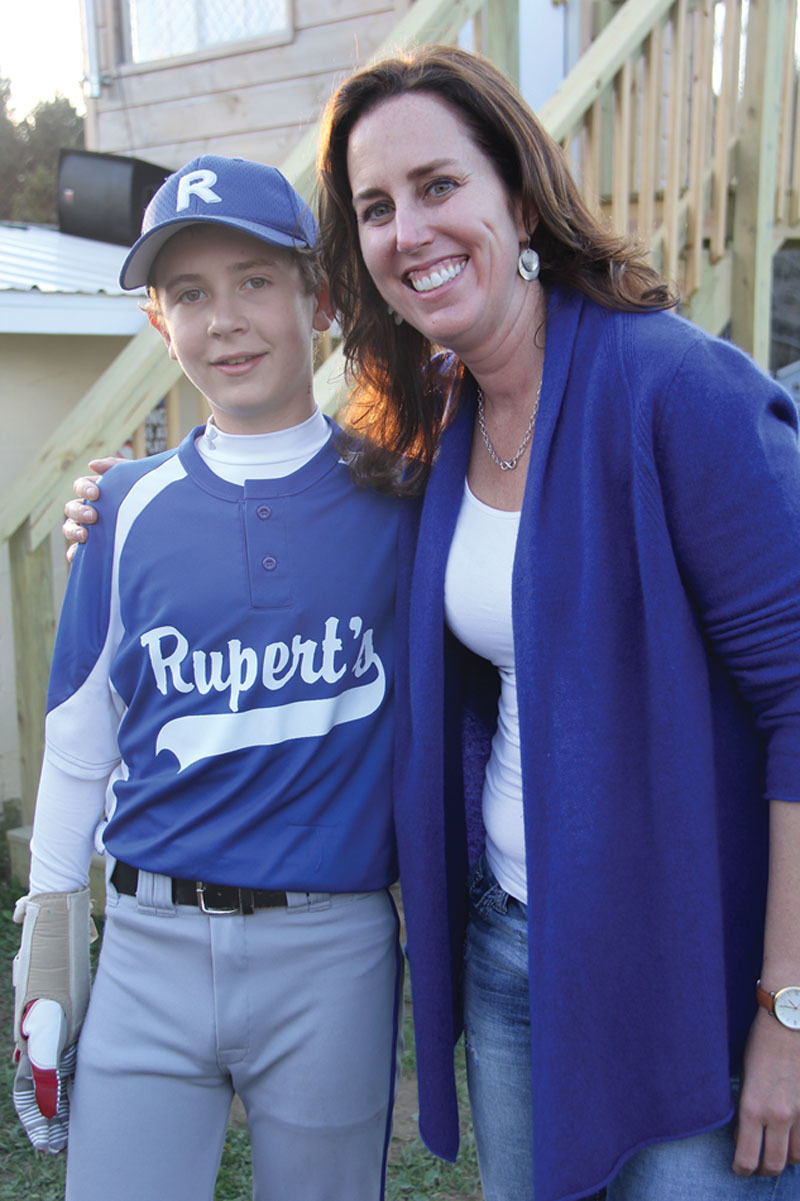
Patient and surgeon before a little league baseball game. Our patient held a special ceremony to recognize all those who cared for him following his injury.

Replantation in the pediatric population, although challenging, can be very rewarding in terms of the outcome (Fig. 4). The lack of underlying comorbidities and the amazing regenerative capacity of children allows surgeons to attempt replantation even in the setting of nerve avulsions, as was the case with our patient.^4^ Despite the contraindication to replant when the nerves are severely stretched, one should attempt replant in a child in this scenario if the child is otherwise stable. Our patient continues to regain function over 2 years out from initial replantation. His shoulder and elbow function are nearly completely normal. He has the potential to undergo innervation free muscle transfer in the future if he does not regain adequate hand function.

## PATIENT OF COURAGE VIDEO

Following a nomination from the senior author L.J.G., the patient detailed in this case was honored by the American Society of Plastic Surgeons as a 2017 Patient of Courage. As part of the ceremony, a special video presentation was made at the Opening Ceremonies of the ASPS’ 2017 Meeting held in Orlando, Fla (**see** video, Supplemental Digital Content 1, which displays the story of Seth Apel. This video is available at the https://www.plasticsurgery.org/patients-of-courage/2017-patients-of-courage).

**Video Graphic 1.**
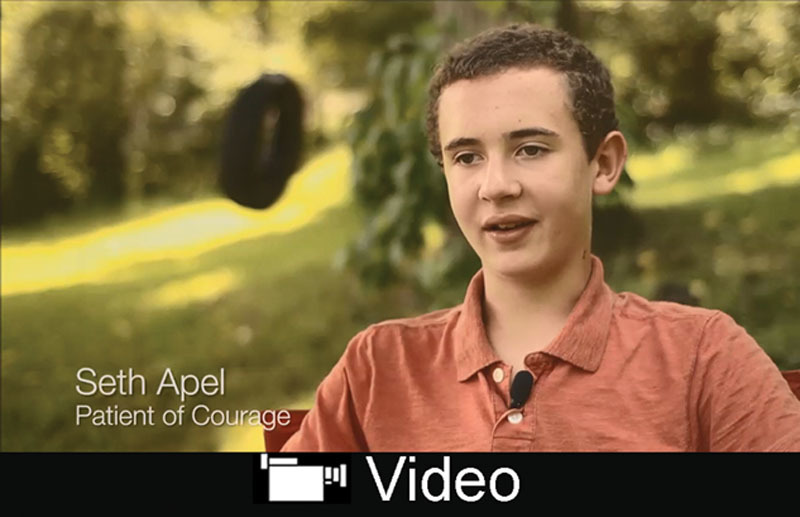
See video, Supplemental Digital Content 1, which displays the story of Seth Apel. This video is available here at https://www.plasticsurgery.org/patients-of-courage/2017-patients-of-courage.

## PATIENT CONSENT

The patient and his family provided written consent for the use of his image.

## Supplementary Material
